# Blocking and reversing hepatic fibrosis in patients with chronic hepatitis B treated by traditional Chinese medicine (tablets of *biejia ruangan* or RGT): study protocol for a randomized controlled trial

**DOI:** 10.1186/1745-6215-15-438

**Published:** 2014-11-10

**Authors:** Jianhui Qu, Zujiang Yu, Qin Li, Yongping Chen, Dedong Xiang, Lin Tan, Chunliang Lei, Wenlin Bai, Hongyan Li, Qinghua Shang, Liang Chen, Xiaoyu Hu, Wei Lu, Zhiqin Li, Da Chen, Xiaodong Wang, Changjiang Zhang, Guangming Xiao, Xun Qi, Jing Chen, Li Zhou, Guofeng Chen, Yonggang Li, Zhen Zeng, Guanghua Rong, Zheng Dong, Yan Chen, Min Lou, Chunping Wang, Yinying Lu, Cuihong Zhang, Yongping Yang

**Affiliations:** Center of Therapeutic Research for Liver Cancer, the 302 hospital of PLA, 100 Xisi Huan Middle Road, Beijing, 100039 China; Liver Disease Department, Fuyang No 2 People’s Hospital, Fuyang, Anhui Province 236015 China; Therapeutic Center for Liver Disease, 88th Hospital of PLA, Taian, Shandong Province 271000 China; Department of Infectious Disease, First Affiliated Hospital of Zhengzhou University, Zhengzhou, Henan Province 450052 China; Fuzhou Infectious Diseases Hospital, Fuzhou, Fujian Province 350025 China; Department of Infectious and Liver Diseases, Liver Research Center, First Affiliated Hospital of Wenzhou Medical University, Wenzhou, Zhejiang Province 325000 China; Department of Infectious Diseases, Southwest Hospital, Third Military Medical University, Chongqing, 400038 China; Guangzhou No 8 People’s Hospital, Guangzhou, Guangdong Province 510060 China; Department of Hepatic Diseases, Shanghai Public Health Clinical Center, Shanghai, 201508 China; National Integrative Medicine Clinical Base for Infectious Diseases, Department of Infectious Diseases, Affiliated Hospital of Chengdu University of Traditional Chinese Medicine, Chengdu, Sichuan Province 610072 China; Tianjin Second People’s Hospital, Tianjin Institute of Hepatology, Tianjin, 300192 China

**Keywords:** Compound *biejia ruangan* tablet, multicenter randomized controlled trial, chronic hepatitis B, hepatocellular carcinoma, hepatic fibrosis

## Abstract

**Background:**

Chronic hepatitis B (CHB) can progress to cirrhosis, hepatocellular carcinoma (HCC) and ultimately liver-related death. Although oral antiviral therapy for patients with CHB reduces the risk of such complications, once cirrhosis is established, the benefits of antiviral therapy are not robustly demonstrated. According to traditional Chinese medicine (TCM), some Chinese herbal medicines promote blood circulation and soften hard masses, and therefore they may block and reverse hepatic fibrosis. The aim of this study is to evaluate the effects of TCM tablets of the compound *biejia ruangan* (RGT) administered for fibrosis, and entecavir (ETV), on the development of HCC in patients with CHB or hepatitis B virus (HBV)-related compensated cirrhosis.

**Methods/design:**

This multicenter, centrally randomized, double-blind, placebo-controlled, parallel-group study is planned to complete within 5 years. For the study, 1,000 with CHB or HBV-related compensated cirrhosis are randomly assigned in a 1:1 ratio to a treatment group (0.5 mg ETV once daily; 2 g RGT three times daily) or a control group (0.5 mg ETV once daily; 2 g RGT dummy agent three times daily). The primary end points are the development of HCC and liver-related death. Secondary end points include disease progression and overall survival.

**Discussion:**

Although antiviral therapy can achieve sustained suppression of HBV replication, thereby preventing cirrhosis, patients with CHB treated with nucleos(t)ide analogs (NUCs) retain a higher risk for HCC compared with patients with inactive disease. Although previous clinical trials with RGT have confirmed the efficacy of blocking and reversing hepatic fibrosis in patients with CHB or compensated cirrhosis, the long-term risk for HCC or disease progression in these patients treated with combination of RGT and NUCs compared with NUCs alone is unclear. Therefore, it is necessary to investigate the effects of the RGT blockade and reversal of hepatic fibrosis on the development of HCC in patients with CHB or HBV-related compensated cirrhosis in large, prospective, multicenter, double-blind, randomized, controlled trials in China.

**Trial registration:**

ClinicalTrials.gov Identifier: NCT01965418. Date registered: 17 October 2013

## Background

Hepatitis B virus (HBV) infection is a major health problem worldwide with an estimated 350 million chronic carriers [1], and carriers in China account for 33% of all chronic carriers globally [2, 3]. Although treatment of chronic hepatitis B (CHB) has dramatically improved over the past decade, chronic HBV infection is still the most common underlying cause of hepatocellular carcinoma (HCC), which remains one of the most common cancers worldwide. A large longitudinal epidemiological study of the natural history of patients with chronic HBV infection has shown that baseline HBV DNA level or cirrhosis is an independent predictor for the development of HCC [4].

Although antiviral therapy can theoretically prevent HCC by inhibiting HBV replication and preventing the development of, or even reversing, cirrhosis, in patients with fibrosis or cirrhosis, eradication or suppression of HBV does not remove this risk and occurrence of HCC, but can control the complications and gain time to prepare for liver transplantation [5–7]. Antiviral therapy has minimal side effects and sustained suppression of HBV replication can be achieved, thus preventing such complications. However, patients with CHB treated with nucleos(t)ide analogs (NUCs) retain a higher risk for HCC compared with patients with inactive stage disease [8].

According to traditional Chinese medicine (TCM), Chinese medicines promoting blood circulation and softening hard masses may be appropriate for blocking and reversing hepatic fibrosis in CHB or CHB-related compensated cirrhosis. Over the past decade, the role of TCM in the treatment and prevention of fibrosis has been confirmed by a growing number of experiments and clinical studies [9]. To date, no randomized clinical trial (RCT) has reported whether TCM (tablets of the compound *biejia ruangan*; RGT) administered to block and reverse hepatic fibrosis in CHB or HBV-related compensated cirrhosis decreases the risk of developing HCC. RGT (Inner Mongolia Furui Medical Science Co Ltd, Wulanchabu, China) was the first antifibrotic herb approved by the Chinese Food and Drug Administration (CFDA) for fibrotic liver disease in China. The main components of RGT are: turtle shell, zedoray rhizome, peony root, *Angelica sinensis*, pseudo-ginseng, campanumaea pilosula, *Astragalus*, dried human placenta, plant worms, *Baphicacanthus* root and farsythio (Table 1). RGT can soften hardness to dissipate stagnation, and disperse blood stasis and detoxification, and enhance *qi* (biological substances or activities that preserve life) and blood. Syndromes in Chinese medicine include: (1) weakening of both *qi* and blood, (2) blockage of meridians (circulation channels for *qi*) by blood stasis and (3) generation of dampness and heat (inflammatory pathogens). All composites in this prescription are commonly used clinically for chronic liver disease, to soften hard masses and dissolve stagnation, and at the same time, to secure essence and eliminate evil. The quality of the herbal drugs was controlled as follows. First, as a specific treatment of liver fibrosis and early cirrhosis, RGT was approved by the CFDA in 1999, with the license number Z1999101. Second, all the components of RGT are purchased from designated and certificated herb plantations. None of these herb providers has changed during the past 10 years. Third, the production workshops for RGT are also certified by the Good Manufacturing Practices standard. No serious adverse effects have been reported even though the drug entered the market >15 years ago. Finally, RGT has passed the Good Clinical Practice (GCP) certification by the CFDA, which is the internationally recognized standard for the design, conduct, recording and reporting of clinical trials involving human subjects.

**Table 1:** **The major ingredients of** ***fufang biejia ruangan pian*** **(RGT)**

Chinese phonetic alphabet name	Latin name	English name
*Biejia*	*Carapax trionycis*	Turtle shell
*Ezhu*	*Rhizoma curcumae*	Zedoray rhizome
*Chishao*	*Radix Paeoniae rubra*	Peony root
*Danggui*	*Radix Angelica sinensis*	*Angelica sinensis*
*Sanqi*	*Radix notoginseng*	Pseudo-ginseng
*Dangshen*	*Radix codonopsis*	Campanumaea pilosula
*Huangqi*	*Radix astragali*	Astragalus
*Ziheche*	*Placenta hominis*	Dried human placenta
*Dongchongxiacao*	*Cordyceps sinensis*	Plant worms
*Banlangen*	*Radix isatidis*	Baphicacanthus root
*Lianqiao*	*Fructus forsythiae*	Farsythio

Some clinical trials have demonstrated that RGT can block the development of hepatic fibrosis and reverse early cirrhosis [10]. Other studies have shown that RGT can significantly improve compensated cirrhosis, especially in CHB and early cirrhosis, with a total rate of effectiveness of 81.67%; after a 1-year follow-up, the total rate of efficacy was shown to be 76.85% [11]. Yang *et al*. [12–14] have reported that RGT significantly blocks early liver fibrosis, inhibiting the proliferation of hepatic stellate cells, reducing collagen synthesis and excess deposition in the space of Disse, dissolving and absorbing formed collagen fibers, and inhibiting expression of collagen α2 (I) mRNA. It has also been shown that RGT increases the phagocytic function of macrophages in the abdominal cavity of mice. Zhao *et al*. [15] have reported similar results, and have proposed that RGT has antifibrotic efficacy through interaction with multiple targets that affect hepatic fibrosis. Some studies have confirmed that RGT has better antifibrotic efficacy in compensated cirrhosis [16, 17], especially HBV-related hepatic fibrosis [18].

Although some studies have concluded that RGT can block and reverse hepatic fibrosis in CHB, there are limited data on preventing or delaying the development of HCC in the treatment of CHB or HBV-related compensated cirrhosis. To address this issue, we are conducting a large, prospective, multicenter, centrally randomized, double-blind, controlled trial to establish whether RGT can prevent or delay the development of HCC by blocking and reversing hepatic fibrosis in CHB or HBV-related compensated cirrhosis. The aims of this clinical trial are: (1) to evaluate the effect of combined application of RGT and entecavir (ETV) on development of HCC in patients with CHB or HBV-related compensated cirrhosis and (2) to evaluate the efficacy of combined application of RGT and ETV in preventing or delaying the incidence of decompensated cirrhosis and disease progression in the treatment of patients with CHB or HBV-related compensated cirrhosis.

## Methods/design

### Study design

**Figure 1:**
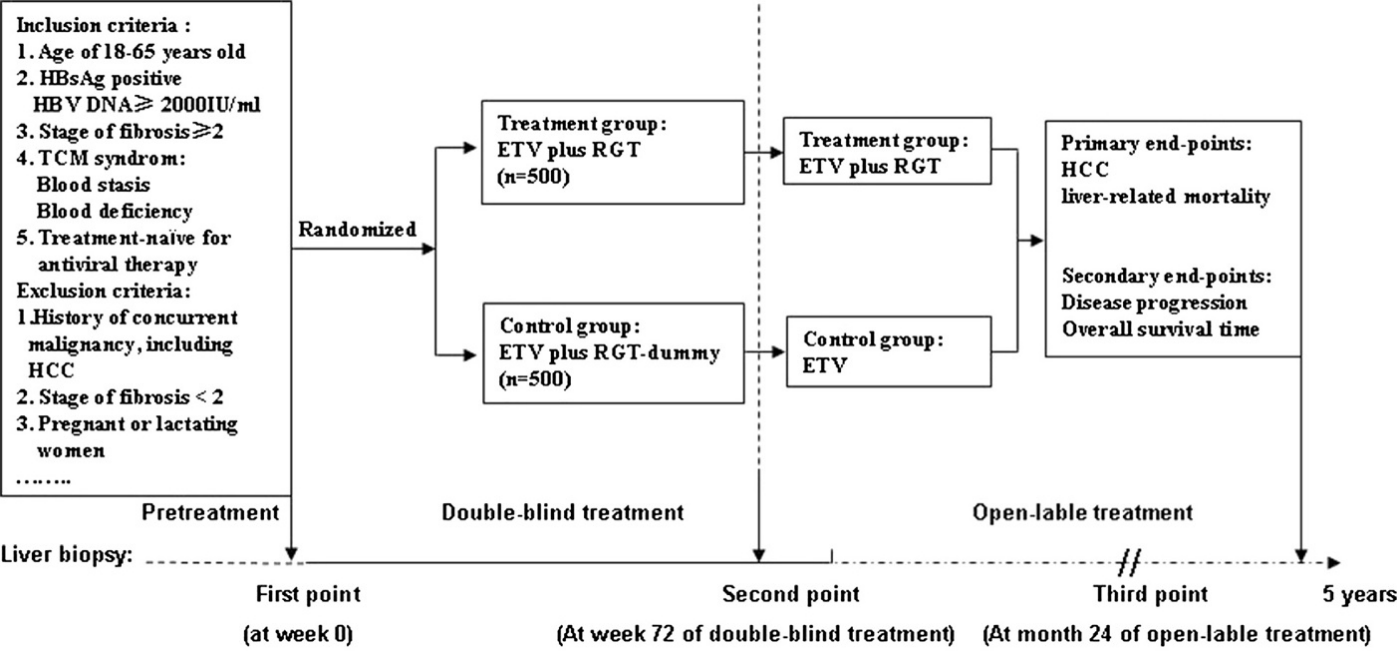
**Flow chart of the current trial.** ETV, entecavir; HBV, hepatitis B virus; HBsAg, hepatitis B surface antigen; HCC, hepatocellular carcinoma; RGT, tablet of compound *biejia ruangan*; TCM, traditional Chinese medicine.

This large, prospective, multicenter, centrally randomized, double-blind, placebo-controlled trial is being conducted in nine institutions between November 2013 and November 2018. The study meets the requirements of the Declaration of Helsinki, and has been approved by the ethics committees of each participating institution. All patients with CHB or HBV-related compensated cirrhosis are carefully screened by a multidisciplinary team to select suitable candidates for this clinical trial, according to the inclusion and exclusion criteria of the present RCT and the patients’ willingness to participate in the study. For the study, 1,000 patients are being randomly assigned in a 1:1 ratio to one of two groups: a treatment group (0.5 mg ETV once daily; 2 g RGT three times daily) or a control group [0.5 mg ETV once daily; 2 g RGT dummy agent (similar in taste, shape and color to RGT; main ingredients include pearl barley and grilled germinate barley) three times daily]. During the double-blind phase, unblinding will take place as soon as sufficient evidence indicates: (1) that the effect of RGT plus ETV blocking and reversing hepatic fibrosis (measured by METAVIR system) is statistically superior to that with ETV alone, (2) that RGT plus ETV does not provide a significant advantage over ETV alone by the second liver biopsy at week 72 of double-blind treatment or (3) safety concerns. Patients who reach an end point are offered open-label RGT plus ETV or ETV alone for 1 year, and patients who have blockade and reversal of hepatic fibrosis are followed up after treatment and have the option to receive RGT plus ETV as an open-label treatment in the event of hepatic fibrosis or necroinflammatory progression. However, patients with stable or reversed disease will continue to receive antiviral therapy with ETV alone, but those with disease progression will receive open-label RGT plus ETV as in the control group. The study will be terminated as soon as sufficient evidence indicates that the outcome of patients with CHB or HBV-related compensated cirrhosis by RGT plus ETV blockade and reversal of hepatic fibrosis is statistically superior to that with ETV alone or does not provide a significant advantage over ETV. If the trial is terminated according to the predefined criteria, patients will be offered open-label treatment for 1 year. Liver biopsies are performed at three defined time points: before the start of randomization (pretreatment), at week 72 of the double-blind treatment, and at month 24 of the open-label treatment in the follow-up study. After 24 weeks of treatment, any patients with a reduction in HBV DNA level from baseline <1 log IU or 2 log copies/mL will be treated with a combination of ETV and adefovir dipivoxil (10 mg once daily) against HBV. The flow chart of the study is shown in Figure 1.

This study is being conducted in accordance with the ethical principles of the Declaration of Helsinki and is consistent with GCP guidelines and applicable local regulatory requirements. Written informed consent is obtained from all randomly assigned patients.

The primary end points in this study are the occurrence of HCC or liver-related mortality. HCC is diagnosed either histologically or clinically based on guidelines proposed by the Chinese Liver Cancer Study Group [19]. According to these criteria, patients are considered positive for HCC if they have one or more risk factors (hepatitis B or C virus infection, or cirrhosis) and one of the following: (1) serum α-fetoprotein (AFP) >400 ng/mL and a positive finding on at least one of three typical imaging studies [dynamic computed tomography (CT), dynamic magnetic resonance imaging (MRI) or hepatic angiography] or (2) serum AFP <400 ng/mL and positive findings on at least two of the three imaging studies. A positive finding for typical HCC on dynamic CT or MRI is defined as increased arterial enhancement followed by decreased enhancement compared with the liver (washout) in the portal or equilibrium phase. Liver-related mortality is defined as death caused by hepatic decompensation (regardless of etiology) or HCC.

Secondary end points are disease progression and overall survival rate. Disease progression is defined as either histological progression reflected by an increase in METAVIR score of at least 1 point or clinical progression reflected by the occurrence of decompensated cirrhosis. Decompensated cirrhosis is defined by the development of complications of portal hypertension (ascites, variceal hemorrhage, hepatic encephalopathy or spontaneous bacterial peritonitis) and/or liver insufficiency (jaundice). Following previous studies [20, 21], liver biopsies are collected at three defined time points: before the start of randomization (unless a biopsy has been performed within 3 months of randomization), at week 72 of the double-blind treatment and at month 24 of the open-label treatment during follow-up. Every patient with liver biopsy of at least six portal tracts is included in the study. To ensure the quality of liver tissue specimens for pathological assessment, liver biopsies in all centers is performed using unified 16G liver biopsy needles and a modified Jamshidi Menghini needle (Allegiance Corporation, McGaw Park, IL, USA), and the tissue is fixed in formalin and embedded in paraffin. Two independent third-party pathologists who are unaware of the patients’ treatment assignment, biopsy sequence and clinical outcome will examine all biopsy slides.

Hepatic fibrosis is assessed by the METAVIR system for fibrosis stage and necroinflammatory activity as a hard index [22, 23]. The fibrosis stages are: no fibrosis (F0), mild fibrosis (F1), moderate fibrosis (F2), severe fibrosis (F3) and cirrhosis (F4). Necroinflammatory activity is: no activity (A0) mild activity (A1), moderate activity (A2) and severe activity (A3). Serum fibrosis biomarkers including Fibrotest, aspartate aminotransferase to the platelet ratio index (APRI), liver stiffness spleen diameter to platelet ratio score (LSPS), and FibroMeters and FibroScan are used to assess hepatic fibrosis as a surrogate index [24, 25]. Hard and surrogates indexes are evaluated at all three time points (baseline, week 72 and week 96 after the open label) for each patient. Changes in liver fibrosis from baseline to weeks 72 and weeks 96 after the open label are analyzed on available pooled data using the METAVIR scoring system and the surrogate index.

The contraindications of liver biopsy include coagulopathy, uncooperative patients, impaired mental status, infection of the hepatic bed and extrahepatic biliary obstruction. In addition, patients undergoing a liver biopsy are required to have an international normalized ratio <1.6 and platelet count >60 × 10^9^/L. For analysis of the histological end point, patients who can be evaluated must have adequate baseline biopsy specimens with a METAVIR necroinflammatory or fibrosis score of at least 1. Progression of hepatic fibrosis or necroinflammatory progression is defined as an increase of at least 1 point.

### Follow-up

At baseline, all patients are screened to confirm the absence of HCC using abdominal ultrasonography and laboratory analyses, including AFP level. During the follow-up, all patients are seen at 3- to 6-month intervals. Periodic surveillance is also performed with abdominal ultrasonography and laboratory analyses, including AFP level, every 3 or 6 months to screen for HCC and other complications related to portal hypertension. Patients also undergo endoscopy every 1 or 2 years for screening and follow-up of gastroesophageal varices. HBV DNA, hepatitis B surface antigen (HBsAg), anti-HBs, hepatitis B e antigen, anti-HBe, and routine hematological and biochemical tests are performed. Adverse events that have occurred since the previous visit are recorded, and toxicity is graded according to predefined criteria.

### Participants and eligibility

Inclusion criteria are as follows: (1) men or women aged 18 to 65 years; (2) persistent serum HBsAg for ≥6 months; (3) serum HBV DNA ≥2000 IU/mL or ≥10,000 copies/mL; (4) baseline liver fibrosis (liver biopsy) stage F ≥2 (METAVIR system); (5) TCM syndrome type: blood stasis, or blood deficiency with toxic heat retention and (6) no antiviral or antifibrotic therapy for 6 months. Patients are excluded if they have any of the following at enrollment: (1) history of concurrent malignancy, including HCC; (2) baseline liver fibrosis (liver biopsy) stage F <2 (METAVIR system); (3) immunosuppression (organ transplantation or immunosuppressant use); (4) severe alcoholism; (5) serious uncontrollable heart, kidney, lung, endocrine, blood, metabolic or gastrointestinal primary disease, or mental illness; (6) pregnancy or lactation; (7) allergic constitution or allergic to TCM use; (8) no prescribed medication, poor compliance, incomplete data affecting the efficacy and safety of those judgments; (9) unsuitable for this trial according to the researchers or (10) co-infection with hepatitis C or D virus or HIV.

### Recruitment

Nine first-class hospitals across the Eastern, Western, Southern, Northern and Central areas of China are involved in this clinical trial. Participants are recruited via posters at each participating center.

### Randomization

This study has two arms. Once enrolled, participants who meet the inclusion criteria are informed about the study and treatment plan. Written informed consent is obtained when the patient accepts this plan and is willing to participate. A consecutively numbered envelope is unsealed by a research assistant. The sealed numbers are generated by a computerized random number generator with SAS software by Boao Tong Medical Technology Co Ltd (Beijing, China) and are concealed and disseminated using opaque envelopes. Based on this number, the patient is randomly assigned in a 1:1 ratio to the treatment group (ETV + RGT, *n* = 500) or control group (ETV + RGT-dummy agent, *n* = 500).

### Sample size

We assumed that the rate of hepatic fibrosis reversal would be 39% in the ETV-treated patients at the end of 2 years [26, 27], and 61% in the RGT-treated patients. Using PASS 2002 statistical software (NCSS, Kaysville, UT, USA), we calculated that we need two groups of 186 cases, and each group needs to enroll 223 cases if there is a drop-out rate of 15%, that is, a total of 446 cases. Currently, up to 45% of all enrolled patients will be accepted for a second liver biopsy. A total of 500 cases is required in each group to achieve a power of 0.80 and a significance level of 0.05 in assessing the difference between the two groups.

### Safety

All adverse events, regardless of their possible association with the disease or study treatment, are recorded. Adverse events are considered to be serious if the investigator determines that they jeopardize the patient, are life-threatening, or could result in hospitalization, disability or death.

### Data and safety monitoring board

To promote consistent study execution at each participating center, independent monitoring visits are performed to supervise trial progress, ensuring that it is conducted, recorded and reported in accordance with the protocol, standard operating procedures, GCP and the applicable regulatory requirements.

The Data and Safety Monitoring Board consists of three independent hepatologists who are not members of the end-point committee and an independent statistician. The board protects the ethical interests and safety of the patients by reviewing interim analyses. The board is empowered to recommend termination of the study on the basis of safety concerns or as soon as sufficient evidence indicates that RGT plus ETV is statistically superior to ETV alone, or that RGT plus ETV does not provide a significant advantage over ETV alone.

### Ethics

The study protocol, written informed consent and posters were approved by the 302nd Military Hospital Ethics Review Committee (2013145D), and were accepted by the ethics committees at each of the institutions involved, and have been performed according to the Declaration of Helsinki.

The names of all ethical bodies in the various centers involved are the 302nd Military Hospital Ethics Review Committee, Fuyang No 2 People’s Hospital Ethics Review Committee, 88th Hospital of PLA Ethics Review Committee, First Affiliated Hospital of Zhengzhou University Ethics Review Committee, Fuzhou Infectious Diseases Hospital Ethics Review Committee, First Affiliated Hospital of Wenzhou Medical University Ethics Review Committee, First Affiliated Hospital of Third Military Medical University Ethics Review Committee, Guangzhou No 8 People’s Hospital Ethics Review Committee, Shanghai Public Health Clinical Center Ethics Review Committee, Affiliated Hospital of Chengdu University of Traditional Chinese Medicine Ethics Review Committee and Tianjin Second People’s Hospital Ethics Review Committee.

### Statistical methods

The randomized subjects who have taken at least one dose of study drug and are evaluated for at least once curative effect after taking medication, constitute the full analysis set and safety analysis set. Those who complete all the visits comprise the per-protocol set. The statistical analysis will be performed using SAS 9.1 software (SAS Institute, Cary, NC, USA). The overall loss rate due to adverse events of each group will be calculated. When subjects enter the trial, they are assessed for demographic data, baseline condition and other essential information and are compared between groups. Cumulative incidence of HCC and overall survival rate will be determined by Kaplan–Meier analysis, and the log-rank test will be used for treatment comparisons. Changes from baseline in liver histology (METAVIR score) as well as in liver and spleen imaging (type B ultrasound) between the two groups will be compared by the Cochran–Mantel–Haenszel test. Changes from baseline in serum markers for liver fibrosis and liver function, transient electrograph (FibroScan) and TCM syndrome between the two groups will be compared by a covariance or non-parametric test. The statistical significance is defined as a two-sided *P* <0.05.

## Discussion

HBV is a common infection that is associated with a considerable burden of liver-related morbidity and mortality worldwide [28]. HBV is strongly associated with HCC by its presence in tumor cells and by the role of persistent HBV infection as a risk factor for the development of HCC [29]. Recent advances have seen the introduction of drug therapy with the potential to have a significant impact on the incidence of liver-related complications [28]. In particular, treatment of CHB has dramatically improved over the past decade [30–32], but persistent HBV infection is still the most common underlying cause of HCC, which remains one of the most common cancers worldwide [33]. Cirrhosis and persistently high viremia are both important risk factors for HCC in patients with CHB [4, 33, 34]. Therefore, antiviral therapy could theoretically prevent HCC by inhibiting HBV replication and preventing the development of, or even reversing, cirrhosis [30–32]. Antiviral therapy alone does not remove this risk and occurrence of HCC [7]. However, it has not been demonstrated whether a combination of TCM and antiviral therapy could have a role in preventing or delaying the development of HCC in patients with CHB.

Clinical trials have been carried out to evaluate the role and efficacy of RGT for HBV-related hepatic fibrosis and compensated cirrhosis [10, 15–18]. RGT administered to block and reverse hepatic fibrosis in CHB or HBV-related compensated cirrhosis is considered one of the treatment options for patients with hepatic fibrosis. Only limited data are available about the effects of RGT blockade and reversal of hepatic fibrosis on the development of HCC in patients with CHB. To date, there have been no RCTs comparing the effects of combined application of RGT and NUCs with NUCs alone on the development of HCC in patients with CHB or HBV-related compensated cirrhosis.

The present clinical trial introduces promising and classical herbs and formulae into the treatment of hepatitis-B-related hepatic fibrosis and compensated cirrhosis. Above all, this clinical study evaluates for patients with CHB or HBV-related compensated cirrhosis: (1) the efficacy of TCM (RGT) in preventing or delaying the occurrence of HCC, (2) the efficacy of RGT in reducing the development of decompensated cirrhosis and disease progression for patients with CHB or HBV-related compensated cirrhosis and (3) whether TCM treatment improves the hepatic microenvironment and reduces the development of HCC.

In summary, the results of the present trial are of value because they will provide an important evidence base for the effects of TCM blockade and reversal of hepatic fibrosis on development of HCC for patients with CHB or HBV-related compensated cirrhosis.

## Trial status

Patient recruitment for this trial is ongoing. Data collection will continue until the end of November 2018.

## Abbreviations

AFP: α-fetoprotein
APRI: aspartate aminotransferase to the platelet ratio index
CFDA: Chinese Food and Drug Administration
CHB: chronic hepatitis B
CT: computed tomography
ETV: entecavir
GCP: Good Clinical Practice
HBsAg: hepatitis B surface antigen
HBV: hepatitis B virus
HCC: hepatocellular carcinoma
LSPS: liver stiffness spleen diameter to platelet ratio score
MRI: magnetic resonance imaging
NUC: nucleos(t)ide analog
RCT: randomized clinical trial
RGT: tablet of compound *biejia ruangan*
TCM: traditional Chinese medicine.
